# Approaches to Enhancing and Sustaining Engagement in Post-Retirement Work: A Scoping Review

**DOI:** 10.1177/08982643251378213

**Published:** 2025-09-26

**Authors:** Raihana Premji, Bao-Zhu Stephanie Long, Kishana Balakrishnar, Aishvinigaa Sathananthan, Charlene Choi, Alexia M. Haritos, Beatrice Yuen, Maryna Mazur, Behdin Nowrouzi-Kia

**Affiliations:** 1 Department of Occupational Science and Occupational Therapy, Temerty Faculty of Medicine, University of Toronto, ON, Canada; 2 Krembil Research Institute-University Health Network, Toronto, ON, Canada; 3 Centre for Research in Occupational Safety & Health, Laurentian University, Sudbury, ON, Canada; 4 Institute for Mental Health Policy Research, Centre for Addiction and Mental Health, Toronto, ON, Canada

**Keywords:** post-retirement work, older adults, health and well-being, financial factors, social welfare

## Abstract

**Background and Objectives:** With an aging population and growing economic hardship for many older adults, post-retirement work is increasingly common but often challenging due to complex, intersecting factors. This scoping review aims to identify key barriers and facilitators to post-retirement work. **Methods:** We searched APA PsycINFO, Embase, CINAHL, Scopus, and Web of Science for peer-reviewed studies (2000–2025) on post-retirement work among adults aged 50–80. Eligible studies underwent two rounds of screening and risk of bias assessment. **Results:** Fifty-two studies were included in this scoping review. Four key themes emerged: (1) social factors, (2) health-related factors, (3) workplace factors, and (4) financial factors. Barriers and facilitators included health status, discrimination, job conditions, financial stability, policy support, social networks, and personal fulfillment. **Discussion:** Post-retirement work is shaped by diverse factors with important policy implications. Future research should examine underrepresented groups and regional differences.

## Introduction

Population aging is a global phenomenon that is impacting health and social systems worldwide (Khan et al., 2024). The number of people in the 60+ age group will rise from 1 billion in 2020 to 1.4 billion by 2030, making up one in six of the world’s population (Ageing and Health, 2024). By 2050, there will be 2.1 billion individuals worldwide aged 60 years or older, and 426 million people aged 80 years or older (Ageing and Health, 2024). Aging is often associated with age-related diseases that can lead to a decline in both physical and mental abilities, thereby limiting the capacity to perform daily activities (Dang et al., 2022). This decline can have a profound effect on notable life changes such as retirement.

Retirement systems differ across the globe, highlighting potential differences in the retirement experience internationally. In Canada, the typical retirement age is 65, at which point individuals begin receiving pension payments from the Government of Canada (CPP Retirement pension. How Much you Could Receive, 2025). These payments can vary in amount based on the age an individual retires, the level of contribution an individual made to the Canandian Pension Plan in their lifetime, and their average lifetime income (CPP Retirement pension. How Much you Could Receive, 2025). In the United States (U.S.), the official age of retirement is between 66 and 67, where individuals are incentivized to work up to 70 years of age to maximize their retirement benefits (See your Full Retirement Age, 2025). In Australia, individuals must be 67 years old to receive pension payments, and many may not be eligible depending on their income-level and assets (Age Pension: Who Can Get It, 2024). These factors can have notable implications for the experience of retirement, and subsequently the likelihood to engage in post-retirement work across countries.

Retirement provides the opportunity for improved health by leaving behind high-stress and high-risk occupations (d’Errico et al., 2022). While retirement is initially linked to increased subjective well-being and life satisfaction, these benefits tend to decline over time (Bound & Waidmann, 2007; Horner, 2014), with many retirees eventually facing reduced mental health and cognitive functioning (Clarke et al., 2012; Falba et al., 2009; Reis & Gold, 1993). Work, for instance, offers a sense of social role and identity (Froidevaux et al., 2018) and is a source of purpose (Ward & King, 2017). When retirees leave the workforce, they often experience a loss of identity tied to their job titles and responsibilities. Many also miss the demands, challenges, and recognition associated with their work (Schaap et al., 2018). Retirement often leads to social isolation and loneliness (Ibrahim et al., 2024). Workplaces provide a natural social environment, and retirement can result in the loss of daily interactions and networks (Segel-Karpas et al., 2018), negatively impacting mental health and overall well-being (Dang et al., 2022). Financial inadequacy is another contributing factor to reduced well-being, as retirement income often falls short of supporting a desired lifestyle (Growing Older in America: The Health & Retirement Study, 2007; Ibrahim et al., 2024). Beyond losing wages, retirees in some countries may also lose access to employer-provided benefits such as health insurance, which can make it difficult to afford healthcare services (Dang et al., 2022). A U.S. survey by Tipirneni et al. (2019) revealed that nearly half of the older adults who did not qualify for low-premium healthcare coverage through Medicare were unable to afford health insurance during retirement (Tipirneni et al., 2019).

These retirement outcomes vary across different groups, with specific demographic factors influencing individuals’ ability to adapt to life after work (Viceisza et al., 2022; Vigezzi et al., 2025). For example, a study by Vigezzi et al. (2025) found that individuals with higher socioeconomic status (SES) are more likely to experience improved health and increased physical activity during retirement. In contrast, those with lower SES tend to experience declines in physical and mental health, increased sedentary behavior, and greater risk of adverse cardiovascular outcomes. Furthermore, a review by Viceisza et al. (2022) examining racial and ethnic disparities in retirement outcomes reported that Black and Hispanic individuals feel less prepared for retirement than their white counterparts. One explanation for this disparity is that white individuals tend to possess higher levels of financial literacy and greater financial assets, enabling more effective retirement planning and reducing concerns about income loss (Viceisza et al., 2022).

Research indicates that psychosocial interventions can mitigate the negative effects of retirement, thereby enhancing older adults’ quality of life and life satisfaction (La Rue et al., 2024; Qorbani et al., 2024; Steffens et al., 2016). For instance, La Rue et al. (2024) found that individuals who were members of social groups reported better adjustment to retirement, higher life satisfaction, and improved mental health compared to those who were not. Similarly, Steffens et al. (2016) demonstrated that participation in social groups were associated with an improved quality of life and a reduced mortality rate following retirement. Additionally, a quasi-experimental study by Qorbani et al. (2024) revealed that participants involved in support groups were less likely to experience feelings of helplessness, failure, idleness, confusion, and inner conflict.

In addition to psychosocial interventions, post-retirement employment offers another avenue for addressing the adverse effects associated with retirement. Post-retirement work refers to any form of paid employment undertaken by individuals after they have officially retired from their primary career or reached the age of retirement (Yan et al., 2023). This can be done in a full-time or part-time basis and can include contract-based work or self-employment. Post-retirement work is essential for addressing the negative aspects of retirement, enabling improved mental well-being (Dingemans & Henkens, 2015; Patel, 2018), financial stability, and continued economic contribution. For instance, Patel (2018) found that elderly participants who engaged in post-retirement work had higher psychological well-being than those who were not involved in post-retirement work. Similarly, Wu et al. (2005) found that post-retirement work leads to increased self-efficacy and enhanced life satisfaction. These improvements in mental health and well-being are apparent since post-retirement alleviates many challenges associated with retirement, such as social isolation, a reduced sense of purpose, and financial insecurity. Post-retirement work also provides opportunities for older adults to earn a wage, thereby addressing their financial instability. According to Statistics Canada, 38% of respondents have returned to paid employment after retirement due to financial considerations (Schellenberg et al., 2005), which in turn tremendously benefits the economy.

Employers, however, often hold negative perceptions about hiring older workers. In Europe and North America, there is a tendency to invest in training for younger and educated workers, rather than older individuals (Brunello, 2001; Brunello & Medio, 2001; Giraud, 2002). Some employers may also view retirement as a well-deserved period of rest, believing that older workers should now step back from the workforce, having already “paid their dues” (Henkens, 2000). Others assume that older workers are less productive and therefore, less valuable to the organization (Van Dalen et al., 2009). However, there are exceptions where some employers recognize that older adults can be just as capable and productive as their younger counterparts and acknowledge their potential to help address the growing labor market shortages (Van Dalen et al., 2009).

Given these benefits, there is a clear trend of increasing numbers of individuals pursuing post-retirement work. Many older adults continue working primarily for financial reasons—such as covering essential expenses, supporting adult children, managing mortgage debt, and addressing pension ineligibility—and financial considerations often shape the timing of retirement (NICE Ageing in Canada, 2025). According to Statistics Canada, 21.8% of Canadians aged 55–59 years and 44.9% of those aged 60–64 years were either wholly or partially retired (Majority of People Planning to Retire Would Continue Working Longer If They Could Reduce Their Hours and Stress, 2023). Among those planning to retire, approximately 55.1% reported that they would continue working part-time, while 48.9% stated they would work fewer hours if it did not affect their pension (Majority of People Planning to Retire Would Continue Working Longer If They Could Reduce Their Hours and Stress, 2023).

Many retirees shift into different fields, such as self-employment or part-time service roles, rather than returning to previous full-time positions. Data show that among workers aged 65 to 69, nearly four in ten (39%) work part-time—32% of men and 51% of women—compared to just 12% of “core-age” workers aged 25 to 54 (MacEwen, 2012). Furthermore, about 40% of workers over 65 are self-employed (MacEwen, 2012). Among retirees who had fully exited the workforce, 12.9% later returned to part-time employment, while only 6.3% returned to full-time work, highlighting that part-time re-entry is considerably more common than a full-time return (Maestas, 2010). Studies, however, show that the lag between retirement and re-entry varies, with some retirees engaging in bridge employment within months of retirement (Davis, 2003), while others returning after several years to supplement income or maintain social engagement (Luke et al., 2016; Maestas, 2010).

Despite the importance of understanding the factors influencing individuals’ decisions to engage in post-retirement employment, a gap remains in supporting retirees transitioning back to work and those actively involved in post-retirement employment. While some studies have discussed the influence of work on the transition to retirement (Eagers et al., 2018, 2019; Sullivan & Al Ariss, 2019), and a few studies have examined motivational drivers of post-retirement work (Hansson et al., 2022; Mykletun, 2015), no studies have comprehensively explored the facilitators and barriers to post-retirement work in a single review.

This scoping review aims to understand the factors that facilitate and hinder the return to work for retirees. This baseline understanding will enable us to first, understand how we can better support retirees during this process and second, how to attract retirees to rejoin the workforce more effectively. The knowledge gathered in this scoping review will provide evidence-based recommendations for policymakers, employers, and support organizations to create more inclusive and supportive work environments for retirees. Additionally, the findings will help shape targeted interventions to address the challenges retirees face when considering or re-entering employment, ensuring a smoother transition and enhancing their overall well-being and productivity.

## Materials and Methods

### Study Design and Registration

A scoping review will be undertaken to consolidate the existing research on retirees’ transition processes and their involvement in post-retirement work. To date, no knowledge synthesis has explored the specific facilitators and barriers to retirees returning to work. The review was conducted following the PRISMA-ScR (Preferred Reporting Items for Systematic reviews and Meta-Analyses extension for Scoping Reviews) (Tricco et al., 2018), with the checklist completed to assist in the development of this manuscript (see Figure 1 in Appendix A). This study is registered with Open Science Framework (https://doi.org/10.17605/OSF.IO/A2JQE).

### Search Strategy

The literature search was carried out across five databases: APA PsycINFO (Ovid), Embase (Ovid), CINAHL (Ebsco), Scopus (Elsevier), and Web of Science (Clarivate). A preliminary search was conducted on APA PsycINFO (see Table 1), and the databases were searched on October 22, 2024. A supplementary search of the reference lists and hand-searching was performed.

**Table 1.** Sample search strategy employed for APA PsycINFO

Line #	Syntax
1	[title-abs-key (bridge employment) OR title-abs-key (unretirement) OR title-abs-key (post retirement work) OR title-abs-key (work after retirement) OR title-abs-key (return to work) OR title-abs-key (work retention) OR title-abs-key (retention rates) OR title-abs-key (bridge employees) OR title-abs-key (re-enter the workforce) OR title-abs-key (post retirement) OR title-abs-key (hiring retirees) OR title-abs-key (age barriers) OR title-abs-key (reintegration) OR title-abs-key (financial security)]
2	[title-abs-key (retiree) OR title-abs-key (older worker) OR title-abs-key (senior) OR title-abs-key (elderly) OR title-abs-key (older adults) OR title-abs-key (working retirees) OR title-abs-key (retired adults) OR title-abs-key (employed retirees) OR title-abs-key (older retirees) OR title-abs-key (old-age pensioner) OR title-abs-key (pensioners)]
3	[(limit-to (DOCTYPE, “ar”) AND (limit-to (PUB YEAR, >1999) AND (PUB YEAR, <2024)) AND (limit-to (LANGUAGE, “english”))]

### Inclusion and Exclusion Criteria

The definition of older adults varies widely in the literature, typically ranging from 50 to 80 years of age and beyond. For this study, we defined an older worker as anyone employed between the ages of 50 and 80 or older to ensure a broader definition. The research team classified the inclusion and exclusion criteria based on the Population, Concept, and Context (PCC) framework (see Table 2).

**Table 2.** Population, Concept and Context framework

PCC framework	Inclusion criteria	Exclusion criteria
Population	Older adults and/or retirees aged 50 to 80 and above, who are currently employed or have the desire to return to work	<50 years of age or 50+ but unemployed or have no desire to return-to-work
Concept	Data encompassing reasons, facilitators, and barriers related to return-to-work and/or employment	Exploring factors related to the barriers and facilitators to return-to-work beyond the scope of post-retirement work
Context	Peer-reviewed academic sources	Systematic reviews, meta-analyses, knowledge syntheses, commentaries, letters to the editor, and non-peer reviewed articles
	Articles published in English language	Non-English language publications
	Articles published from 2000 and onwards	Articles published before 2000

### Data Collection

Five reviewers independently conducted a thorough literature search to identify relevant studies based on predefined inclusion and exclusion criteria using Covidence (Covidence, 2024), a collaborative software tool for literature reviews. The process involved multiple stages, including an initial title and abstract review followed by a full-text review. At each stage, any disagreements or uncertainties regarding study eligibility were resolved through discussions between the reviewers, with a final consensus reached through mutual agreement or consultation with the senior author (BNK). This iterative approach ensured consistency and thoroughness in screening all the selected studies. Inter-rater reliability was regularly evaluated during the screening and review processes to measure the degree of agreement between the five reviewers. To quantify agreement and ensure consistency in study selection, tools such as Cohen’s kappa coefficient were used (McHugh, 2012). Kappa values ranged from 0.41 to 0.60, indicating moderate inter-rater reliability for full-text screening, with a considerably high proportion of agreements (between 50% and 86%).

### Data Extraction

After the screening and review stages were completed, data were extracted from the included studies. Five reviewers worked independently to extract data from the included papers in the scoping review using a developed and approved template in Microsoft Excel (Microsoft, 2025). A pilot test was conducted prior to the complete data extraction process to ensure inter-rater agreement among the first five studies that met the inclusion and exclusion criteria. The data charting template encompassed the following categories: (1) study title; (2) author name(s); (3) publication year; (4) country of origin; (5) study design; (6) descriptions of the population (sample size, gender, age, and illness); (7) data collection tool(s); (8) return-to-work facilitators; and (9) return-to-work barriers. Drawing on the findings of these categories helped create the theme table. If the data is unclear or incomplete, the study authors will provide clarification upon request from senior author (BNK).

### Data Synthesis

Five independent reviewers collaborated to identify the overarching themes that they deemed offered the most comprehensive summary of the literature. Using Braun and Clarke’s (2006) framework to guide theme development, a descriptive thematic analysis was conducted (Braun & Clarke, 2006). This six-step process involved reviewing the data, generating initial code ideas, and systematically coding the significant items. Codes were then grouped into potential themes, and relevant data were gathered for each. The themes were validated through comparison with coded extracts and the full dataset, followed by further refinement. This approach ensured the validity and robustness of the themes.

## Results

A total of 1,589 research articles were initially screened based on their titles and abstracts. Of these, 1,314 articles were excluded because they did not meet the inclusion criteria, leaving 275 for full-text review. After completing the full-text review in Covidence, 52 studies were selected for inclusion. Seven studies were identified via hand-searching, which all met the eligibility criteria. We also reviewed the reference lists of the included studies but found no additional articles to include for data extraction. A total of 59 studies were included in the review. The detailed process is illustrated in Figure 1.

**Figure 1.**
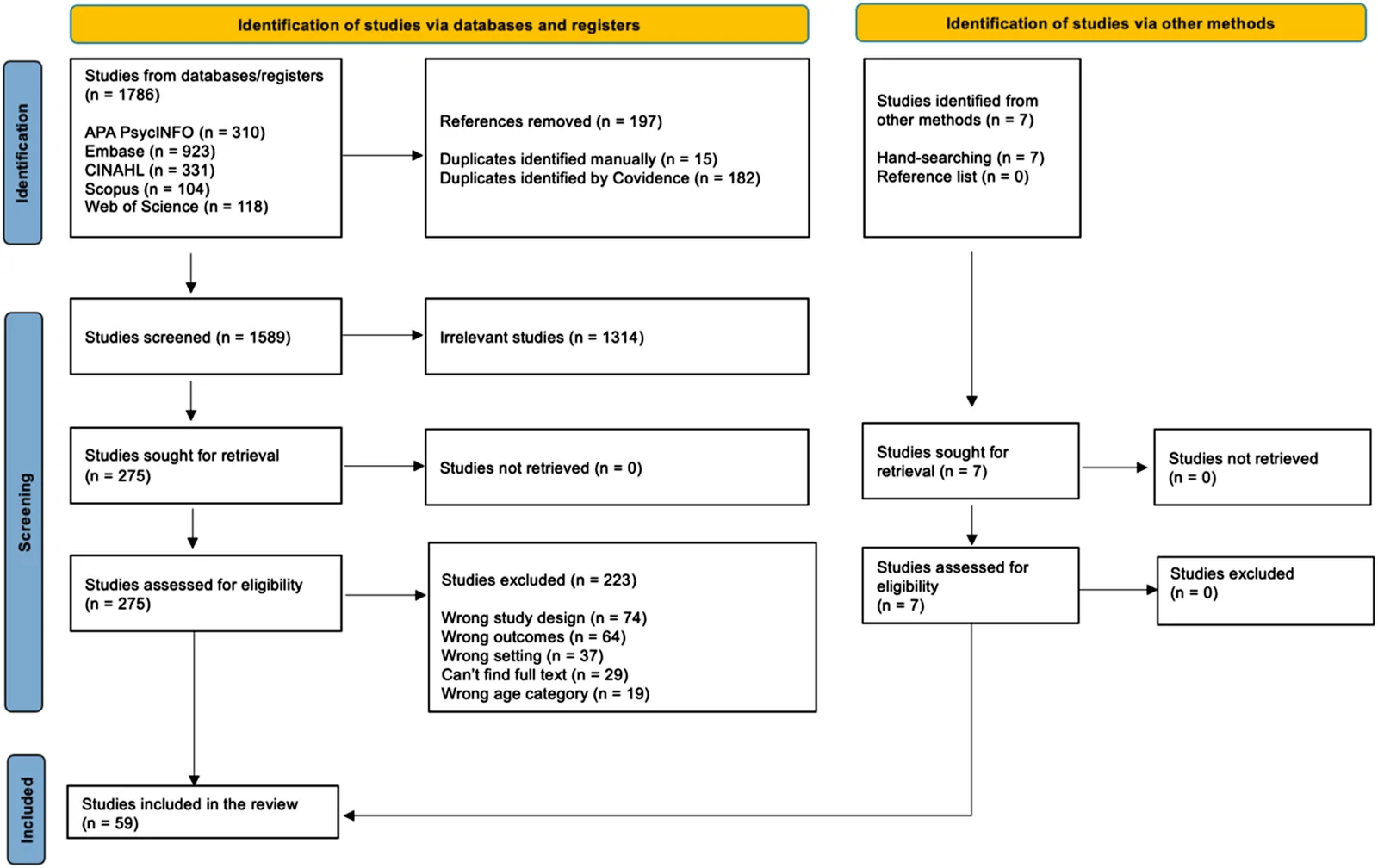
PRISMA Table

### Study Characteristics

The 59 included studies were conducted in various countries, including the following, ordered by number of included studies: United States, the Netherlands, Sweden, Germany, Canada, Denmark, Australia, Iran, Japan, South Korea, Thailand, Finland, Brazil, Israel, Taiwan, Austria, Belgium, Czech Republic, France, Italy, Hungary, Poland, Spain, Estonia, Portugal, Slovenia, England, Switzerland, and Greece. Among these studies, 42 quantitative studies (cross-sectional, longitudinal, cohort) provided an overview of the factors influencing retirees’ decisions to return to work, including demographic, economic, and health-related variables; 12 qualitative studies delved into retirees’ personal experiences, motivations, and perceptions about returning to work; three mixed-methods studies examined both the broader statistical trends and retirees’ personal narratives regarding their transition back into the workforce; and one was a quasi-experimental study offering insights into the effectiveness of different interventions or programs designed to support retirees’ reintegration into the workforce. The detailed characteristics of the included studies are shown in Table 3 (Appendix A). The sample participants of the articles mostly consisted of males and females (*n* = 49), with the majority of the articles having a higher percentage of males (*n* = 28). Some studies did not report gender breakdown (*n* = 9). See Table 4 (Appendix A) for a detailed gender breakdown of each included study (*n* = 59).

### Overarching Themes

Figure 2 presents a summary of the main overarching themes and sub-themes that were inductively derived, including (1) social, (2) health-related, (3) workplace, and (4) financial factors. See Table 5 (Appendix A) for barriers and Table 6 (Appendix A) for facilitators, along with a detailed outline of the studies included in each theme.

**Figure 2.**
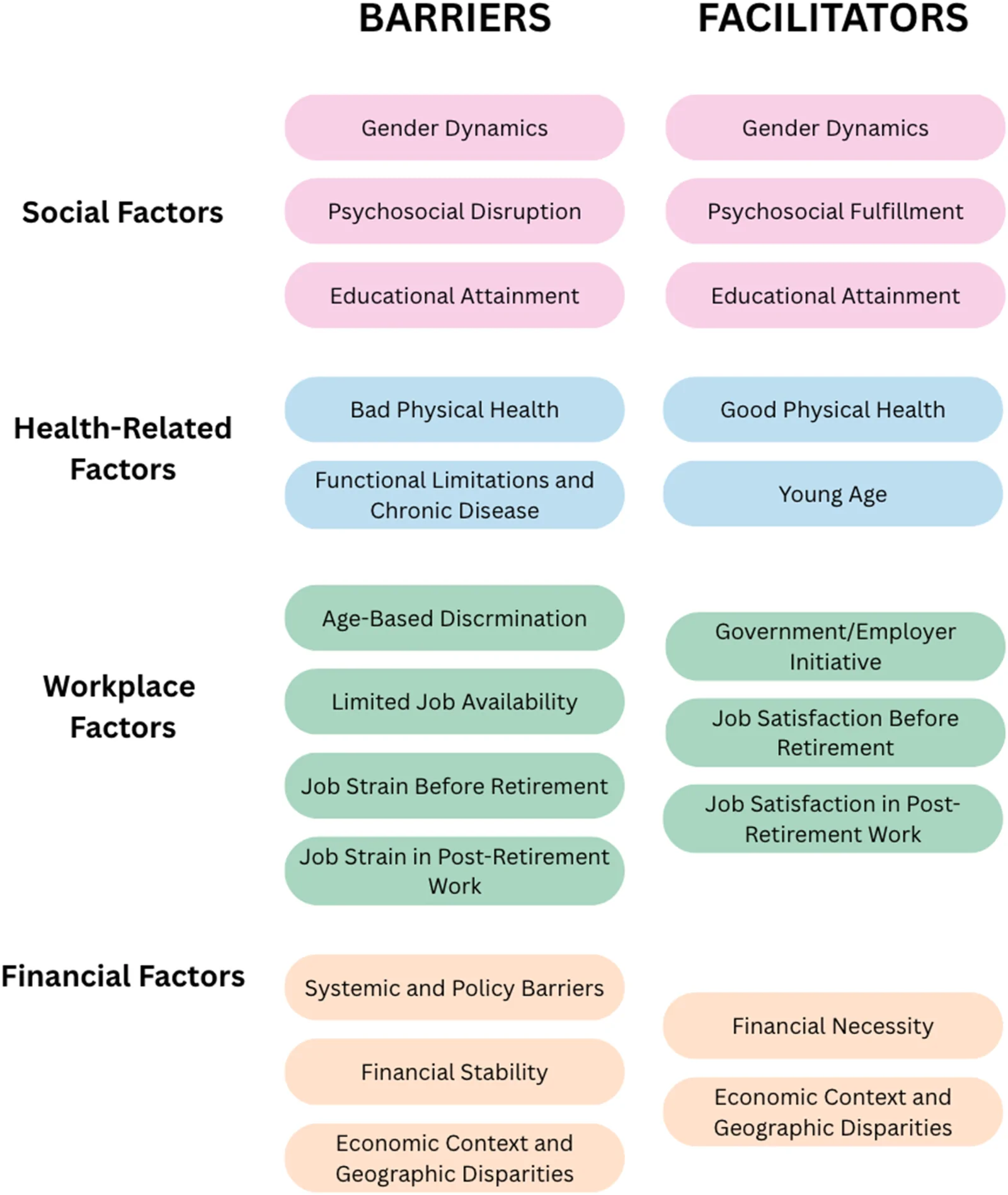
Concept Map of Identified Barriers and Facilitators to Post-Retirement Work. *Note*. Four Main Themes (Social Factors, Health-Related Factors, Workplace Factors, and Financial Factors) are Depicted Above and Organized by Color. Sub-themes on the Left are Categorized as Barriers, while Sub-themes on the Right are Categorized as Facilitators

### Barriers to Post-retirement Work

#### Social Factors

##### Gender Dynamics

Seventeen studies identified gender dynamics as a critical barrier to post-retirement work. The general trend seems to be that men are more likely than women to engage in post-retirement work (Basakha et al., 2022; Boot et al., 2016; De Wind et al., 2016; Gonzales & Nowell, 2017; Homaie Rad et al., 2017; Kail & Warner, 2013; Mermin et al., 2007; Pleau & Shauman, 2013). Several studies have reported on the likely causes of this disparity. For instance, Armstrong-Stassen and Staats (2012) found that retired women are more likely than their male counterparts to feel obsolete and unqualified to return to work after retirement, which may discourage re-entry and reduce retention among female retirees (Armstrong-Stassen & Staats, 2012). Interestingly, Venneberg and Wilkinson (2008) found that working male retirees also struggle with reintegration into the workforce, with reports of having “zero power” and feeling the need to “rebuild their reputation” (Venneberg & Wilkinson, 2008). This demonstrates the differences in the perspectives of women and men who engage in post-retirement work. The greatest motivation for women to engage in post-retirement work appears to be financial necessity, with many working female retirees having dependents or being without a spouse (Armstrong-Stassen & Staats, 2012; Bennett et al., 2016; Dingemans & Möhring, 2019; Pleau & Shauman, 2013; Weber et al., 2019).

Among working female retirees, many were found to have more difficulty with work–life balance when engaging in post-retirement work, with reports of higher job strain compared to men (Venneberg & Wilkinson, 2008; Weber et al., 2019). These challenges may be attributed to the unpaid work that women manage through personal and family responsibilities (Venneberg & Wilkinson, 2008; Wu et al., 2024) and the nature of work they prefer. For instance, women are more likely than men to work in jobs with greater emotional demands. To combat this, other studies have found that women are also more likely to work part-time hours or choose self-employment, which may be a way to improve their ability to maintain a work-life balance while engaging in post-retirement work (Kail & Warner, 2013; Kerr & Armstrong-Stassen, 2011; Weber et al., 2019).

##### Psychosocial Disruption

Twenty-four studies identified psychosocial disruption as a critical barrier to post-retirement work. Decision-making in older adults appears to be influenced by several personal, psychological, and social factors that impact their motivation to engage in post-retirement work. First, multiple studies have found that a lack of recognition and appreciation for accomplishments, experience, knowledge, and commitment strongly discourages older workers from prolonging their retirement (Armstrong-Stassen, 2008; Bengtsson & Flisbäck, 2021; Lee, 2022; Palermo et al., 2024). Furthermore, many retirees are concerned about the social perceptions and implications of engaging in post-retirement work (Manor & Holland, 2022; Mazumdar et al., 2021).

Several studies reported traditional gender roles, diminished social status, criticism and guilt for crowding out younger workers, negative perceptions from peers and pressure to meet socially accepted norms surrounding the retired lifestyle as critical psychosocial barriers to engaging in post-retirement work (Bennett et al., 2016; Manor & Holland, 2022; Mazumdar et al., 2021; Platts et al., 2023). Conversely, many retirees seem to embrace the socially accepted lifestyle of retirement, preferring to spend time with grandchildren or engage in self-discovery (Dingemans et al., 2016; Fasbender et al., 2014).

Fasbender et al. (2014) found that many retirees report a desire for self-discovery and growth, and hesitate to engage in post-retirement work, fearing that they may disrupt their personal growth (Fasbender et al., 2014). Additionally, Lee (2022) found that the death of a loved one can also trigger a shift in perspectives among older adults, leading them to prioritize reduced workloads and more time with family (Lee, 2022); ultimately reducing their desire to engage in post-retirement work. Among retirees who engage in post-retirement work, many report feelings of anxiety and lack of self-confidence as barriers to satisfaction and retention in post-retirement work (Luke et al., 2016; Ulrich & Brott, 2005).

#### Health-Related Factors

##### Functional Limitations and Chronic Disease

Sixteen studies identified functional limitations and chronic diseases as critical barriers to post-retirement work. The general trend is that older adults with chronic health conditions, musculoskeletal discomfort, poor self-rated health, and other work-related disabilities are less likely to engage in post-retirement work (Andersen et al., 2020; Basakha et al., 2022; Deller et al., 2009; Dingemans et al., 2016; Dingemans & Henkens, 2015; Doan et al., 2014; Kerr & Armstrong-Stassen, 2011; Mermin et al., 2007; Platts et al., 2023; Pleau & Shauman, 2013; Thomassen et al., 2022; Ulrich & Brott, 2005; Zhan et al., 2009). Dingemans and Henkens (2015) found that 13% of retirees were forced to retire due to health-related problems, indicating that while there may still be a desire to work, physical health is a critical barrier to post-retirement work (Dingemans & Henkens, 2015).

Two studies also cited emotional challenges related to transitioning back to work and feelings of incompetence as impacting mental health and, subsequently, job satisfaction and retention among working retirees (Anderson & Guo, 2018; Ulrich & Brott, 2005). Lowden et al. (2021) also found that being retired for an extended period could impact re-entry into the workforce, with sedentary and mentally passive lifestyles leading to health conditions, such as sleep problems and cognitive decline, which limit one's ability to effectively engage in post-retirement work (Lowden et al., 2021). Finally, Shacklock and Fulop (2007) found that perceived poor health, both by oneself and one’s peers, can also limit opportunities for post-retirement work (Shacklock et al., 2007).

#### Workplace Factors

##### Age-Based Discrimination and Limited Job Availability

Fourteen studies identified age-based discrimination and 13 studies identified limited job availability as a critical barrier to post-retirement work. Implicit and explicit ageism were common themes that emerged from the included studies, which reported reduced job prospects due to age-related biases and stereotypes (Gonzales & Nowell, 2017; Kail & Warner, 2013; Platts et al., 2023; Shacklock et al., 2007; Zhan et al., 2009). Older adults report such discrimination during the hiring process and even afterward, with many struggling to work cohesively with their younger co-workers, finding differences in work ethic and skills to be particularly challenging (Luke et al., 2016; Massingham & Chandrakumara, 2019; Mazumdar et al., 2021; Oude Mulders et al., 2014). For example, Mazumdar et al. (2021) found that older workers were less likely to be hired in technology-driven fields due to being perceived as “past their prime” (Mazumdar et al., 2021); a sentiment shared by many young employers and employees (Ulrich & Brott, 2005; Yang, 2012). Conversely, many older workers may be perceived as overqualified for some work, which reduces their likelihood of being hired and limits their job satisfaction in post-retirement work if they are not in their preferred working environment (Mazumdar et al., 2021; Shacklock et al., 2007).

Several studies have also shown that older workers struggle with finding suitable employment after retirement due to a mismatch of skills, high unemployment rates and limited availability of jobs that are otherwise suitable for older workers (Dingemans & Henkens, 2019; Kerr & Armstrong-Stassen, 2011; Mizuochi, 2024; Pleau & Shauman, 2013; Ulrich & Brott, 2005; Yang, 2012). Finally, many older workers report experiencing explicit disrespect and devaluation, which significantly reduces their desire and likelihood of engaging in post-retirement work (Armstrong-Stassen, 2008; Lee, 2022; Luke et al., 2016; Ulrich & Brott, 2005; Venneberg & Wilkinson, 2008).

##### Job Strain Before Retirement and Job Strain in Post-Retirement Work

Twenty-three studies identified job strain as a critical barrier to post-retirement work. Older workers reported that job strain before retirement and job strain in post-retirement work were equally discouraging and likely responsible for the reduced desire to engage in post-retirement work. For example, Karen et al. (2006) found that retirees who held professional positions before retirement were less likely to return to work after retirement (Karen et al., 2006); likely due to burnout and occupational stress in previous roles. Several studies show that the lack of human resources practices targeted for older workers, high psychological distress, high workload, physically demanding work, and limited job flexibility and autonomy in previous jobs decreased their likelihood of engaging in post-retirement work (Armstrong-Stassen, 2008; Bennett et al., 2016; Deller et al., 2009; Kail & Warner, 2013; Thomassen et al., 2022). It was also shown that older workers are discouraged to engage in post-retirement work when they were underappreciated at previous jobs (Lee, 2022).

Many studies have also reported job strain in post-retirement work as a significant barrier to retention among older adults who have returned to work after retirement. Such job strain seems to result from a lack of flexibility and autonomy, high physical job demands, limited ability for physical work, working at a slower pace, and requiring new skills (Berglund et al., 2017; Doan et al., 2014; Kaewpan et al., 2023; Karpinska et al., 2013; Luke et al., 2016; Mazumdar et al., 2021; Palermo et al., 2024; Rau & Adams, 2005; Weber et al., 2019). These factors make it challenging for older workers to reintegrate into the workforce, which can lead to tension in the workplace (Luke et al., 2016). Several studies also discuss the lack of training and resources available to older workers to acquire the skills necessary for post-retirement work, which can create job strain and is often beyond their control (Ulrich & Brott, 2005; Venneberg & Wilkinson, 2008). Furthermore, differences in work ethic between older and younger workers can lead to conflict and hinder effective teamwork (Massingham & Chandrakumara, 2019).

### Financial Factors

#### Systemic and Policy Barriers and Financial Stability

Fourteen studies identified systemic and policy barriers as critical barriers to post-retirement work. The included studies show that many retirees have little incentive to engage in post-retirement work because of pension payments, social security, and employer-sponsored or private health insurance, which are factors that provide financial stability (Andersen et al., 2020; Basakha et al., 2022; Gonzales & Nowell, 2017; Kail & Warner, 2013; Karen et al., 2006). Several included studies have shown that financial stability is a critical barrier to post-retirement work, rendering wealthy or high-income household members less likely to engage in post-retirement work (Basakha et al., 2022; Gonzales & Nowell, 2017; Mermin et al., 2007; Palermo et al., 2024; Pleau & Shauman, 2013; T.-K. Wu et al., 2024). However, many retirees wish to engage in post-retirement work but face other systemic and policy barriers. For example, Mazumdar et al. (2021) discussed policies that prevent retirees from benefiting from post-retirement work by limiting the number of hours permitted for work and making pension payments contingent on restricted post-retirement activities (Mazumdar et al., 2021). Similar barriers include lower wages for older workers, mandatory retirement policies, and a lack of formal policies to support reintegration into the workforce after retirement (Mizuochi, 2024; Oude Mulders et al., 2014; Platts et al., 2023; Pleau & Shauman, 2013; Venneberg & Wilkinson, 2008; Yang, 2012).

#### Economic Context and Geographic Disparities

Four studies identified the economic context and geographic disparities as critical barriers to post-retirement work. Post-retirement work seems to differ across countries because of their different economic contexts (Dingemans & Henkens, 2019; Dingemans & Möhring, 2019). For example, Dingemans and Henkens (2019) found that post-retirement work was less common in countries of the Global North despite low pension income, due to stronger social support, healthcare systems, and labor markets (Dingemans & Henkens, 2019). Homaie Rad et al. (2017) also found differences in local geographic regions, with retirees from urban areas being less likely to engage in post-retirement work than their rural counterparts (Homaie Rad et al., 2017).

### Facilitators to Post-Retirement Work

#### Social Factors

##### Professional Networks and Educational Attainment

Six studies identified a professional network as a critical facilitator of post-retirement work. Active employment not only serves as a means of financial support but also provides opportunities for social engagement and exposure. Withdrawal from work due to retirement can lead to social isolation, which many retirees combat by engaging with their community and spending time with friends (Gonzales & Nowell, 2017; Venneberg & Wilkinson, 2008). Several studies have found that retirees were able to build, maintain, and rely on strong social and professional networks as facilitators for post-retirement work (Gonzales & Nowell, 2017; Oude Mulders et al., 2014; Venneberg & Wilkinson, 2008; Weber et al., 2019; T.-K. Wu et al., 2024; Yang, 2012).

Seven studies identified educational attainment as a facilitator of post-retirement work. Three articles found that higher educational attainment was associated with a higher likelihood of engaging in post-retirement work and higher job satisfaction among workers (Dingemans & Henkens, 2019; Horiguchi, 2024; Mermin et al., 2007; Pleau & Shauman, 2013). Conversely, two studies found that lower educational attainment increases the likelihood of engaging in post-retirement work and experiencing high job strain (Basakha et al., 2022; Dingemans & Henkens, 2020).

##### Psychosocial Fulfillment

Thirty-five studies identified psychosocial fulfillment as a critical facilitator of post-retirement work. A common theme across most of the included studies was the social and psychological struggles that retirees faced when transitioning out of the workforce. Many older retirees report struggling with social isolation and declining mental health because of their withdrawal (Fasbender et al., 2014; Horiguchi, 2024; Kaewpan et al., 2023; Shacklock et al., 2007; Venneberg & Wilkinson, 2008; Weber et al., 2019; Zhan et al., 2009). Many retirees engage in post-retirement work in response to these challenges. For example, working retirees have reported engaging in post-retirement work to feel part of a community and endorse social support from co-workers as a facilitator of satisfaction and retention (Björklund Carlstedt et al., 2022; Brown et al., 2014; Deller et al., 2009; Dendinger et al., 2005; Dingemans & Henkens, 2015; Gonzales & Nowell, 2017; Horiguchi, 2024; Kim, 2021; Mazumdar et al., 2021; Shacklock et al., 2007; Ulrich & Brott, 2005; Venneberg & Wilkinson, 2008; T.-K. Wu et al., 2024; Zhan et al., 2009).

Many retirees also engage in post-retirement work to strengthen and maintain their sense of identity, purpose, and structure, to feel a sense of fulfillment, and to engage in meaningful work in their daily lives (Anderson & Guo, 2018; Fasbender et al., 2015; Kail & Warner, 2013; Kerr & Armstrong-Stassen, 2011; Kim, 2021; Luke et al., 2016; Manor & Holland, 2022; Mazumdar et al., 2021; Oude Mulders et al., 2014; Platts et al., 2023; Shacklock et al., 2007; Ulrich & Brott, 2005; Venneberg & Wilkinson, 2008; Weber et al., 2019; Yang, 2012; Zhan et al., 2009). Lastly, retirees also reported engaging in post-retirement work out of desire for personal growth, increased contribution to society, mentorship opportunities, and for proactive health maintenance (Armstrong-Stassen & Staats, 2012; Deller et al., 2009; Dendinger et al., 2005; Dingemans & Henkens, 2015; Fasbender et al., 2014; Kerr & Armstrong-Stassen, 2011; Kim, 2021; Lee, 2022; Luke et al., 2016; Manor & Holland, 2022; Mazumdar et al., 2021; Oude Mulders et al., 2014; Shacklock et al., 2007; Venneberg & Wilkinson, 2008; T.-K. Wu et al., 2024; Yang, 2012).

#### Health-Related Factors

##### Good Physical Health and Young Age

Eight studies identified good physical health as a critical facilitator of post-retirement employment. The health status of retired individuals is a strongly influential factor in post-retirement work for employers and employees. Older workers with higher self-reported health and perceived good health are more likely to be hired and maintain longevity in the workforce post-retirement (Boot et al., 2016; De Wind et al., 2016; Fasbender et al., 2014; Kail & Warner, 2013; Karpinska et al., 2011; Kerr & Armstrong-Stassen, 2011; Lowden et al., 2021; Mermin et al., 2007; Pleau & Shauman, 2013; Thomassen et al., 2022; T.-K. Wu et al., 2024; Zaccagni et al., 2024; Zhan et al., 2009).

Twelve studies identified young age as a critical facilitator of post-retirement work. The general trend among the included studies seems to be that younger workers are more likely to engage in post-retirement work, likely due to greater physical mobility, perceived health, and longevity (Basakha et al., 2022; Brown et al., 2014; Dingemans & Möhring, 2019; Kail & Warner, 2013; T.-K. Wu et al., 2024; Zaccagni et al., 2024). Conversely, four studies found that older workers were more likely to be hired (De Wind et al., 2016; Dingemans et al., 2016; Karpinska et al., 2011, 2013). Karpinska (2011) reported that this may be particularly true during labor shortages (Karpinska et al., 2011).

#### Workplace Factors

##### Government/Employer Initiatives

Twelve studies identified government and employer initiatives as critical facilitators of post-retirement work. Several studies report government support in increasing engagement in post-retirement work by creating more jobs as well as enforcing policy reforms to pension qualifications and mandated retirement, allowing more flexibility for older workers to prolong retirement (Andersen et al., 2020; Mermin et al., 2007; Mizuochi, 2024; Pleau & Shauman, 2013; Thomassen et al., 2022; Yang, 2012). Employer initiatives have also been shown to encourage post-retirement work by implementing HR practices that ensure the valuation and respect of older workers as well as fostering healthy and compatible work environments for their reintegration, satisfaction, and retention (Armstrong-Stassen, 2008; Burkert & Hochfellner, 2017; Horiguchi, 2024; Karpinska et al., 2013; Mizuochi, 2024; Pleau & Shauman, 2013; Rau & Adams, 2005).

##### Job Satisfaction Before Retirement and Job Satisfaction in Post-Retirement Work

Four studies identified job satisfaction before retirement as a critical facilitator of post-retirement work. Retirees seem to value flexibility, autonomy, and social status, as the presence of these factors in work before retirement was shown to improve the likelihood of engaging in post-retirement work (Burkert & Hochfellner, 2017; Dingemans et al., 2016; Dingemans & Möhring, 2019; T.-K. Wu et al., 2024).

Twenty-seven studies identified job satisfaction in post-retirement work as a critical facilitator of job satisfaction and retention. Many older workers have reported flexibility, autonomy, and equity as the major facilitators of satisfaction and retention in post-retirement work (Armstrong-Stassen, 2008; Armstrong-Stassen & Staats, 2012; Berglund et al., 2017; Dingemans & Möhring, 2019; Kaewpan et al., 2023; Karpinska et al., 2011, 2013; Kerr & Armstrong-Stassen, 2011; Kim, 2021; Luke et al., 2016; Manor & Holland, 2022; Mazumdar et al., 2021; Mermin et al., 2007; Platts et al., 2023; Rau & Adams, 2005; Shacklock et al., 2007; Thomassen et al., 2022; Ulrich & Brott, 2005; Venneberg & Wilkinson, 2008; Weber et al., 2019; Yang, 2012; Zhan et al., 2009). Older workers also value reduced working hours, equal opportunities for training and development, social support, reduced job demands, high work engagement, and interesting and challenging work (Armstrong-Stassen, 2008; Armstrong-Stassen & Staats, 2012; Brown et al., 2014; De Wind et al., 2016; Dingemans & Möhring, 2019; Karpinska et al., 2013; Lee, 2022).

#### Financial Factors

##### Financial Necessity

Twenty-one studies identified financial necessity as a critical facilitator of post-retirement work. Many retirees return to work for financial support, with many requiring supplemental income due to low lifetime income, low pension income, benefit reductions, inability to afford health insurance, debt and supporting dependents (Armstrong-Stassen & Staats, 2012; Basakha et al., 2022; Björklund Carlstedt et al., 2022; Burkert & Hochfellner, 2017; Burkhalter et al., 2022; De Wind et al., 2016; Deller et al., 2009; Dingemans & Henkens, 2015, 2020; Hansson et al., 2022; Homaie Rad et al., 2017; Kail & Warner, 2013; Karen et al., 2006; Leinonen et al., 2020; Mizuochi, 2024; Pleau & Shauman, 2013; Venneberg & Wilkinson, 2008; Zaccagni et al., 2024). In some cases, the extent of financial needs might be so severe that older workers are forced to settle for unfavorable conditions, increasing the likelihood of job strain (Dingemans & Henkens, 2020; Weber et al., 2019). Bennet et al. (2016) found that some retirees who endorse traditional gender roles are forced to disregard them because of the extent of their financial needs (Bennett et al., 2016), demonstrating that financial necessity can be a strong facilitator, regardless of other potential barriers that exist among certain groups of retirees.

##### Sub-Themes as Both Barriers and Facilitators to Post-Retirement Work

In this review, we conducted an inductive analysis to derive themes and sub-themes given the most prominent patterns that emerged across the included studies. While individual studies presented themes as either barriers or facilitators to post-retirement work, our synthesis revealed that certain themes could play both roles depending on the study context—some themes emerged as barriers in one set of studies and as facilitators in others. In this section, we will highlight the themes with dual roles, as an extension to our primary findings.

The gender breakdown of studies indicated that women were more likely to experience barriers in returning to work than men due to occupational stress (e.g., poor job autonomy) and mental fatigue; therefore, men were more likely to return to work. However, both men and women were more likely to return to work if they had the option of a flexible work schedule to accommodate their leisure activities. This indicates that flexibility in work arrangements is important for both genders when deciding to return to work, but holds greater significance for women. Five studies found that women were more likely than men to engage in post-retirement work because they have dependents, prioritize financial stability, and have a greater desire to share their knowledge, highlighting gender dynamics as a facilitator of post-retirement work (Armstrong-Stassen & Staats, 2012; Bennett et al., 2016; Burkert & Hochfellner, 2017; Dingemans & Möhring, 2019; Karen et al., 2006).

Marital status was also found to have a dual role as both a barrier and a facilitator to post-retirement work (*n* = 25). For example, retirees who are divorced or widowed are more likely to participate in post-retirement work than those who are married or remarried after divorce (Björklund Carlstedt et al., 2022; Dingemans & Möhring, 2019; Leinonen et al., 2020; Pleau & Shauman, 2013). Women who stayed divorced had a higher probability of working, whereas those who remarried following divorce were not statistically different from their continuously married counterparts. Moreover, compared to married retirees, those who were divorced were more likely to work in bridging positions and remain employed. On the other hand, those who had never been married, divorced, or widowed were more likely to continue working in a low-stress position until retirement.

Two studies found that educational attainment is a barrier to post-retirement work, with many retirees who hold university degrees reporting financial stability and having other means of engagement, rendering post-retirement work redundant (Basakha et al., 2022; Gonzales & Nowell, 2017). Furthermore, two studies identified the economic context and geographic disparities as facilitators of post-retirement work. Retirees in less wealthy countries are more likely to engage in post-retirement work due to reduced pension income, which differs from that in wealthier nations, given economic differences (Dingemans & Henkens, 2019, 2020). Additionally, several studies identified physical health as a barrier to post-retirement work, reporting poor self-rated and perceived health and physical limitations as primary barriers to retirement (Andersen et al., 2020; Basakha et al., 2022; Dingemans et al., 2016; Kail & Warner, 2013; Karpinska et al., 2013; Pleau & Shauman, 2013; Thomassen et al., 2022). Finally, Leinonen et al. (2020) identified financial stability as a facilitator of post-retirement work, reporting that retirees with higher incomes are more likely to engage in post-retirement work under more favorable conditions than less wealthy retirees (Leinonen et al., 2020).

## Discussion

Our scoping review examined the key facilitators and barriers influencing retirees’ return to work, enabling the identification of effective strategies to support their transition. By identifying factors that encourage or discourage re-entry, our findings offer guidance on how to better attract and retain retirees in the workforce, as well as help shape targeted interventions to address the challenges they face. Four themes regarding post-retirement work were identified: *social, health-related, workplace, and financial factors*.

### Barriers and Facilitators to Post-Retirement Work

#### Social Factors

Social factors were identified as one of the four major themes that influence post-retirement work. Our findings revealed that gender dynamics and educational attainment served as both barriers and facilitators, while psychosocial disruption was a barrier. Additionally, professional networks and psychosocial fulfillment were found to be both barriers and facilitators.

According to our results, several studies have shown that men were more likely than women to engage in post-retirement work. To conceptualize this factor associated with post-retirement work, we drew up on a systematic review by Galkutė and Herrera (2020). Although their review differed in scope and methodology, comparing our findings with theirs provides a more comprehensive understanding of post-retirement work and highlights areas for tailored interventions. The systematic review reported that four studies identified a negative relationship between being a woman and post-retirement work (Galkutė & Herrera, 2020). However, 11 studies found no such association, highlighting the inconsistency of our findings. This may partly be because many of these studies did not specifically investigate gender as a barrier or facilitator to post-retirement work. In some of the studies, gender was not discussed in the findings, suggesting that its potential role may have been underexplored or underreported. Regardless, based on our findings and the literature, being a woman was commonly seen as a barrier to post-retirement work, while being a man was commonly seen as a facilitator. This may be due to women having more difficulty with work–life balance leading to high job strain compared to men.

However, interestingly, our study found that sometimes women may engage in post-retirement work, but due to specific reasons such as financial necessity, having dependents or no spouse. Therefore, these findings only reflect how gender differences may be influenced by the value individuals place on their work. As a result, our scoping review, which examined not only the subjective meanings of work but also broader contextual factors, contributes to the literature by showing that gender dynamics can function as both barriers and facilitators to post-retirement work. Our study also found that women were more likely to work part-time hours or choose self-employment, suggesting that work can be restructured to offer part-time options and other flexible working arrangements for older adults, especially women, to mitigate the issues involving work–life balance and strain, allowing them to continue working after retirement.

Regarding educational attainment, Galkutė and Herrera (2020) report mixed findings (Galkutė & Herrera, 2020). Three studies found positive associations between higher education and post-retirement work, and two identified negative associations and nine non-significant associations. These findings suggest that the perceived benefits or drawbacks of retirement vary across educational attainment levels. Individuals with higher or lower levels of education may experience retirement differently depending on their personal and contextual circumstances, which can, in turn, either encourage or discourage continued employment. This is consistent with our results as educational attainment was found to be both a facilitator and a barrier, with higher or lower educational attainment in some contexts encouraging post-retirement work while in other contexts higher educational attainment can be a barrier to post-retirement work. According to our results, this may be due to other factors such as financial stability and having other means of engagement when having higher education. However, the literature points out to another explanation which involves geographical variation. Galkutė and Herrera (2020) highlight how socioeconomic development differs across countries, noting that in developed countries, individuals with lower education may still have adequate living conditions (Galkutė & Herrera, 2020). In contrast, in less developed countries, lower education might be more detrimental thereby forcing older adults to continue working (Galkutė & Herrera, 2020). This suggests that since the studies in our scoping review include both individuals from developing and developed countries, this may explain why the results were ambiguous when it came to educational attainment. It also suggests how much of an impact geographical variation has on older adults’ decisions regarding post-retirement employment. In contrast, psychosocial fulfillment was more consistent with the existing literature. Galkutė and Herrera (2020) reported that three studies found positive associations between workplace recognition and post-retirement work (Galkutė & Herrera, 2020).

However, it is important to note that although the systematic review conducted by Galkutė and Herrera (2020) also examined the factors influencing post-retirement work and included studies from various countries, there are key differences in scope and methodology, making our scoping review more comprehensive and a valuable contribution to the existing literature. First, many of the studies included in their review focused more on implicit or organizational factors. In contrast, our review utilized a broader approach by examining studies that also focus on social, workplace, financial, and health-related factors, thereby addressing this gap and providing a more comprehensive understanding of the barriers and facilitators. Their review also focused on studies published between 2008 and 2018, while our broader time frame, from 2000 and onwards, allowed us to include over 20 studies published after 2018, identifying more recent literature. Additionally, they included both peer-reviewed and grey literature and examined individuals aged 40 and above, while our scoping review was limited to peer-reviewed articles and individuals aged 50–80. Another important thing to note was that the systematic review’s search syntax did not include terms directly related to retirement or return to work, which may have limited the scope of their findings. In contrast, our search strategy explicitly targeted these terms, resulting in more relevant studies. Therefore, the included studies were not the same as those included in our scoping review.

The current scoping review has also addressed limitations noted in their systematic review by incorporating qualitative and mixed-methods studies and consisted of more studies from different countries, including studies from Asia, North America, and South America. Despite these differences, our findings align with those reported in their review, indicating consistency across a broader range of literature and making the comparison of findings between these two reviews valuable.

#### Health-Related Factors

Health-related factors have emerged as another theme influencing post-retirement work. According to the results, functional limitations and chronic diseases often served as barriers, while good physical health and young age served as facilitators. A systematic review found a negative relationship between functional limitations and post-retirement work in two studies; however, one study found no significant association (Galkutė & Herrera, 2020). This is consistent with the idea that healthier workers are more likely to demonstrate optimal work performance (Harvard Business Review, 2013). They are less likely to take time off, whereas unhealthier workers experience greater productivity losses to both absenteeism and presenteeism (Tryon, 2014).

#### Workplace Factors

Workplace factors were identified as major themes influencing post-retirement work. According to our findings, age-based discrimination, pre-retirement job strain, and post-retirement job strain served as barriers, whereas government/employer initiatives, pre-retirement job satisfaction, and post-retirement job satisfaction served as facilitators. In contrast, limited job availability served as both a barrier and facilitator. Some of these findings are strongly supported by the literature. For instance, the systematic review found that five studies reported a positive association between work flexibility and continued work (Galkutė & Herrera, 2020). Additionally, age-based discrimination and limited job availability have also been seen as barriers for older workers in the literature. Although seniors are willing to re-enter the workforce, a study found that employers often hold negative attitudes towards older workers, often based on stereotypes such as inflexibility and a lack of competence (Thomassen et al., 2020). While this study specifically focused on older unemployed individuals rather than retirees, its findings are still relevant, as these biases could potentially impact retirees’ chances of finding work after retirement.

The results of this study show that workplace factors can play a dual role on post-retirement attitudes depending on individual context. For example, one’s experience in the workplace before and after retirement can strongly encourage or discourage engagement and retention in post-retirement work. Many factors such as age-based discrimination, high job strain, minimal managerial support, and lack of meaningful work can significantly dissuade an individual from engaging in post-retirement in the long-term. Conversely, employers that prioritize inclusive and flexible work environments by valuing and supporting older workers may be more likely to see higher engagement in post-retirement work. Consequently, workplace factors can encourage and discourage older adults from participating in post-retirement work depending on the type of work environment, culture, and practices.

#### Financial Factors

The final major theme identified as influencing post-retirement work was financial. Based on our findings, systemic and policy barriers, as well as financial stability, serve as barriers, while financial necessity acts as a facilitator. Additionally, economic context and geographic disparities are both barriers and facilitators. The results of this study also show that financial factors can act as both barriers and facilitators to post-retirement work. Nuances in eligibility for pension payments and social security systems might cause reduced financial benefits for retirees who are earning an income, which effectively dissuades them from remaining in or re-entering the workforce. Additionally, older workers might find that work-related costs such as transportation can offset their financial benefits from engaging in post-retirement work, further influencing their decision to work. Conversely, many older adults might be forced to work out of financial necessity, perhaps due to insufficient retirement savings. Therefore, financial factors can have both positive and negative effects on the likelihood of engaging in post-retirement work among older adults.

### Limitations

This scoping review had a few limitations that should be acknowledged when interpreting the results. During the inclusion of relevant studies, the review was limited to articles published in the English language, potentially missing valuable findings and diverse perspectives presented in non-English publications. Additionally, the exclusion of relevant grey literature, including unpublished reports, organization documents, and both published and unpublished conference proceedings, may have further limited the scope of the review. These sources could have provided additional context or highlighted other key facilitators and barriers that may not have been presented in the included studies, potentially contributing to a more complete understanding of the range of barriers and facilitators impacting retirees’ return to work.

Additionally, geographical variation was identified as a key limitation in this scoping review. Since the included studies were conducted in various countries, these countries can vary according to retirement age, retirement systems, work environments prior to retirement, and cultural habits related to work such as working hours, cultural expectations, and values regarding the retirement transition and being retired. This can influence which factors are seen as barriers or facilitators to engaging in post-retirement work, making it difficult to draw generalizable conclusions. Moreover, although this review identified numerous barriers (e.g., age-based discrimination, job strain, and health-related limitations) that inhibit post-retirement work, the included studies were largely descriptive and did not quantify the extent to which these barriers reduce participation. Consequently, we cannot estimate the precise increase in return-to-work rates that would result from reducing these barriers. However, the consistency of these barriers across multiple contexts suggests that interventions targeting them—such as enhancing flexibility, providing retraining, and addressing age-related stereotypes—are likely to improve return-to-work outcomes. Future studies with quantitative designs (e.g., longitudinal or intervention studies) are needed to provide robust estimates of the potential increase in return-to-work rates following barrier reduction.

### Future Research and Implications

This scoping review highlights a critical gap in the literature on underrepresented groups. Future research exploring barriers and facilitators of post-retirement work should consider investigating how these factors differ across intersecting identities such as race, gender, immigration status, and disability. For instance, there is limited evidence on how the retirement experiences of immigrant or racialized older workers differ from those of their white counterparts. This lack of evidence underscores a need for more intersectional and equity-informed research among older adults and post-retirement attitudes. Future researchers should also consider expanding their geographical scope to include international data, allowing for a more comprehensive understanding of retirement attitudes on a global scale. Specifically, studies from developing countries remain scarce which limits our understanding of how cultural and economic influences on post-retirement work differ across the world. This critical gap must be addressed to ensure employers and policymakers can design culturally sensitive workplace strategies to address the needs of a diverse and aging workforce and facilitate post-retirement work. Additionally, given how our study examined only paid work, future studies should consider examining the factors associated with engagement in non-paid work among older adults (i.e., volunteering).

The findings of this review indicate that post-retirement work is influenced by a multidimensional set of social, occupational, health-related, and financial factors. These findings call policymakers across these disciplines to implement policies that facilitate post-retirement work among older workers. For example, employers should prioritize strengthening their HR practices to be equitable and supportive of older workers, increase opportunities for flexible and enjoyable work arrangements, such as part-time roles and mentorship opportunities, and foster a positive and inclusive work environment. Additionally, workplaces might also consider creating roles specifically targeted towards older workers that leverage their skills while minimizing physical or psychological strain, thereby creating an optimal work environment that benefits both the employees and employers. Similarly, the government should continue to reduce systemic barriers that prevent older workers from engaging in post-retirement work, such as raising the mandatory retirement age, making pension payments more flexible, and increasing accessibility to health insurance to allow retirees to pursue post-retirement work for fulfillment rather than necessity. Governments might also consider implementing wage subsidies or tax benefits to incentivize employers to hire older workers, further facilitating post-retirement work.

## Conclusion

This scoping review aimed to identify the barriers to and facilitators of engaging in post-retirement work. The findings reveal critical social, occupational, health-related, and financial barriers and facilitators that significantly influence older workers’ ability and desire to engage in post-retirement work. While the current scoping review provides a substantial overview of these critical influences, future research could benefit from expanding the population and geographic limitations to include underrepresented groups on a global scale. The implications of this study call for policymakers across various disciplines to address the highlighted barriers and employers to encourage the identified facilitators in the workplace to improve job satisfaction and longevity in post-retirement work.

## Figures and Tables

**Figure 3.**
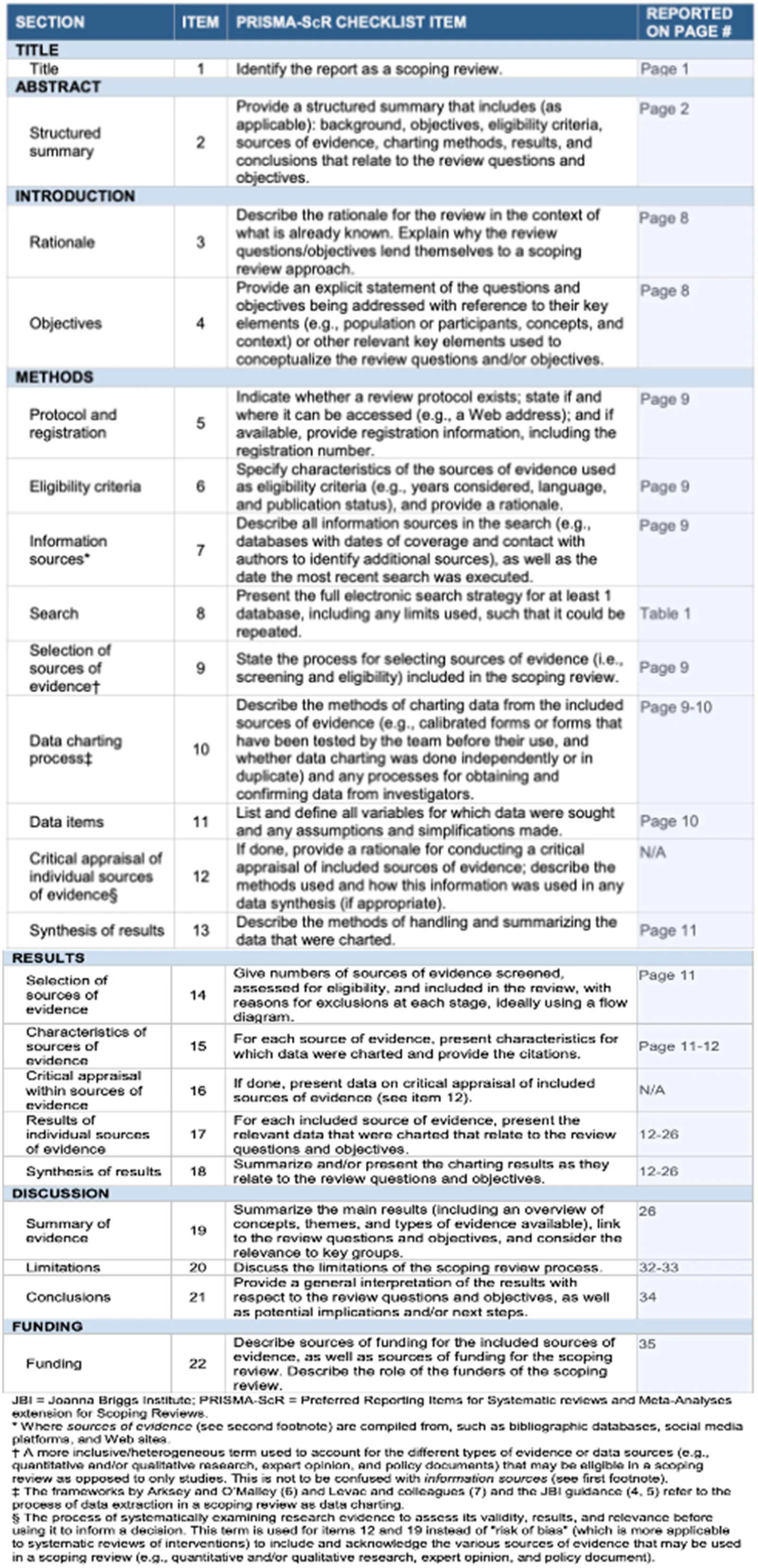
PRISMA-ScR Checklist

## Data Availability

All data generated or analyzed during this study are included in this published article [and its supplementary information files].*

## Appendix A

**Table 3.** Study Characteristics of the Studies that Foster and Encourage Participation in Post-Retirement Work

Author, year	Country	Study design	Sample size
Andersen et al. (2020)	Denmark	Quantitative study	11,444
Anderson and Guo (2018)	United States	Qualitative study	30
Armstrong-Stassen (2008)	Canada	Quantitative study	609
Armstrong-Stassen and Staats (2012)	Canada	Quantitative study	549
Basakha et al. (2022)	Iran	Quantitative study	1,280
Bengtsson and Flisbäck (2021)	Sweden	Qualitative study	15
Bennett et al. (2016)	United States	Quantitative study	482
Berglund et al. (2017)	Sweden	Quantitative study	
Björklund Carlstedt et al. (2022)	Sweden	Quantitative study	1,741
Boot et al. (2016)	Netherlands	Mixed-methods study	256
Brown et al. (2014)	United States	Quantitative study	1,140
Burkert and Hochfellner (2017)	Germany	Quantitative study	4,694
Burkhalter et al. (2022)	Switzerland	Quantitative study	1,241
Deller et al. (2009)	Germany	Mixed-methods study	146
Dendinger et al. (2005)	United States	Quantitative study	108
De Wind et al. (2016)	Netherlands	Quantitative study	1,054
Dingemans and Henkens (2015)	Netherlands	Quantitative study	1,248
Dingemans and Henkens (2020)	11 European countries	Quantitative study	2,926
Dingemans and Henkens (2019)	16 European countries	Quantitative study	54,156
Dingemans et al. (2016)	Netherlands	Quantitative study	1,221
Dingemans and Möhring (2019)	13 European countries	Quantitative study	11,369
Doan et al. (2014)	Canada	Quantitative study	228
Fasbender et al. (2014)	Germany	Quantitative study	551
Fasbender et al. (2015)	Germany	Quantitative study	2,149
Gonzales and Nowell (2017)	United States	Quantitative study	8,334
Gonzales et al. (2017)	United States	Quantitative study	8,334
Hansson et al. (2022)	Sweden	Quantitative study	3,123
Homaie Rad et al. (2017)	Iran	Quantitative study	6,307
Horiguchi (2024)	Japan	Quantitative study	331
Kawepan et al. (2023)	Thailand	Qualitative study	78
Kail and Warner (2013)	United States	Quantitative study	3,873
Karen et al. (2006)	United States	Quantitative study	283
Karpinska et al. (2013)	Netherlands	Quantitative study	238
Karpinska (2011)	Netherlands	Quantitative study	444
Kerr and Armstrong-Stassen (2011)	Canada	Quantitative study	282
Kim (2021)	South Korea	Qualitative study	12
Lee (2022)	United States	Quantitative study	31,127
Leinonen et al. (2020)	Finland	Quantitative study	10,879
Lowden et al. (2021)	Brazil	Quantitative study	10,073
Luke et al. (2016)	Australia	Qualitative study	22
Manor and Holland (2022)	Israel	Qualitative study	28
Massingham and Chandrakumara (2019)	Australia	Mixed-methods study	34
Mazumdar et al. (2021)	Canada	Qualitative study	26
Mermin et al. (2007)	United States	Quantitative study	2145
Mizuochi (2024)	Japan	Quantitative study	11,991
Oude Mulders et al. (2014)	Netherlands	Quantitative study	443
Palermo et al. (2024)	England	Quantitative study	4,436
Platts et al. (2023)	Sweden	Mixed-methods study	25
Pleau and Shauman (2013)	United States	Quantitative study	257,59
Rau & Adams (2005)	United States	Quasi-experimental study	120
Shacklock et al. (2007)	Australia	Qualitative study	20
Tomassen et al. (2022)	Denmark	Quantitative study	30,000
Ulrich and Brott (2005)	United States	Qualitative study	24
Venneberg and Wilkinson (2008)	United States	Qualitative study	12
Weber et al. (2019)	Netherlands	Quantitative study	228
Wu et al. (2024)	Taiwan	Qualitative study	2,981
Yang (2012)	South Korea	Qualitative study	34
Zaccagni et al. (2024)	Denmark	Quantitative study	5,474
Zhan et al. (2009)	United States	Quantitative study	12,189

**Table 4.** Gender Breakdown of Included Studies (*n* = 59)

Author	Gender breakdown		Marital status		
	Women	Men	Married	Not Married/Divorced	Widow
Andersen et al. (2020)	46.7%	53.3%	N/A	N/A	N/A
Anderson and Guo (2018)	30%	70%	N/A	N/A	N/A
Armstrong-Stassen (2008)	40%	60%	79%	21%	N/A
Armstrong-Stassen and Staats (2012)	36.7%	63.2%	76%	N/A	N/A
Basakha et al. (2022)	49.9%	50.1%	70.2%	3.6%	26.2%
Bengtsson and Flisbäck (2021)	60%	40%	N/A	N/A	N/A
Bennett et al. (2016)	33%	67%	75%	9%	11%
Berglund et al. (2017)	N/A	N/A	N/A	N/A	N/A
Björklund Carlstedt et al. (2022)	35%	65%	66%	27%	N/A
Boot et al. (2016)	58.2%	0.418	85.9%	14.1%	N/A
Brown et al. (2014)	51.6%	48.4%	72.7%	N/A	N/A
Burkert and Hochfellner (2017)	59.58%	40.42%	N/A	N/A	N/A
Burkhalter et al. (2022)	42.8%	57.2%	N/A	N/A	N/A
Deller et al. (2009)	31%	69%	N/A	N/A	N/A
Dendinger et al. (2005)	29%	71%	87%	N/A	N/A
De Wind et al. (2016)	38%	62%	N/A	N/A	N/A
Dingemans and Henkens (2015)	25%	75%	N/A	N/A	N/A
Dingemans and Henkens (2020)	47%	53%	71%	17%	12%
Dingemans and Henkens (2019)	53%	47%	N/A	27%	N/A
Dingemans et al. (2016)	25%	75%	N/A	N/A	N/A
Dingemans and Möhring (2019)	46%	54%	11%	15%	N/A
Doan et al. (2014)	41.67%	58.33%	N/A	N/A	N/A
Fasbender et al. (2014)	43.9%	56.1%	N/A	N/A	N/A
Fasbender et al. (2015)	54.4%	45.6%	74.7%	25.3%	N/A
Gonzales and Nowell (2017)	53.75%	46.25%	57%	N/A	N/A
Gonzales and Nowell (2017)	53.8%	46.2%	57%	N/A	N/A
Hansson et al. (2022)	N/A	N/A	N/A	N/A	N/A
Homaie Rad et al. (2017)	N/A	N/A	N/A	N/A	N/A
Horiguchi (2024)	13.9%	13.9%	N/A	N/A	N/A
Kawepan et al. (2023)	N/A	N/A	N/A	N/A	N/A
Kail and Warner (2013)	N/A	N/A	N/A	N/A	N/A
Karen et al. (2006)	N/A	N/A	N/A	N/A	N/A
Karpinska et al. (2013)	23.5%	76.5%	N/A	N/A	N/A
Karpinska et al. (2011)	N/A	N/A	N/A	N/A	N/A
Kerr and Armstrong-Stassen (2011)	55%	45%	62%	N/A	N/A
Kim (2021)	50%	50%	N/A	N/A	N/A
Lee (2022)	52.36%	47.64%	72.46%	25.15%	N/A
Leinonen et al. (2020)	53.23%	46.79%	78.5%	12.2%	2.4%
Lowden et al. (2021)	55%	45%	N/A	N/A	N/A
Luke et al. (2016)	40.9%	59.1%	N/A	N/A	N/A
Manor and Holland (2022)	N/A	NA	100%	N/A	N/A
Massingham and Chandrakumara (2019)	64.71%	35.29%	N/A	N/A	N/A
Mazumdar et al. (2021)	19.2%	80.8%	73%	11.5%	11.5%
Mermin et al. (2007)	50.1%	49.9%	70.8%	29.2%	N/A
Mizuochi (2024)	23.4%	76.6%	N/A	N/A	N/A
Oude Mulders et al. (2014)	33.2%	66.8%	N/A	N/A	N/A
Palerno et al. (2024)	100%	0%	67.7%	31.4%	N/A
Platts et al. (2023)	60%	40%	N/A	N/A	N/A
Pleau and Shauman (2013)	63.8%	36.2%	61.7%	6.7%	26.9%
Rau & Adams (2005)	30%	70%	80.8%	N/A	N/A
Shacklock et al. (2007)	50%	50%	85%	10%	5%
Tomassen et al. (2022)	54.5%	45.5%	N/A	N/A	N/A
Ulrich and Brott (2005)	46%	54%	71%	13%	8%
Venneberg and Wilkinson (2008)	41.7%	58.3%	N/A	N/A	N/A
Weber et al. (2019)	25.87%	74.12%	75.43%	11.84%	N/A
Wu et al. (2024)	42.94%	57.06%	82.4%	17.6%	N/A
Yang (2012)	N/A	N/A	N/A	N/A	N/A
Zaccagni et al. (2024)	21.8%	78.2%	82.2%	17.8%	N/A
Zhan et al. (2009)	57%	43%	76.8%	N/A	N/A

**Table 5.** Summary of the Major Themes and Sub-Themes for Barriers to Post-Retirement Work

Themes	Sub-themes	Number of studies	Total sample size
Social factors	Gender dynamics	17	69, 888
	Psychosocial disruption	24	79, 853
Health-related factors	Functional limitations and chronic disease	16	96, 114
Workplace factors	Age-based discrimination	14	53, 182
	Job strain before retirement	8	69, 501
	Job strain in post-retirement work	15	21, 358
	Limited job availability	13	108, 779
Financial factors	Systemic and policy barriers	14	117, 942
	Financial stability	9	49, 116
	Economic context and geographic disparities	4	74, 758

**Table 6.** Summary of the Major Themes and Sub-Themes for Facilitators to Post-Retirement Work

Themes	Sub-themes	Number of studies	Total sample size
Social factors	Professional networks	6	12, 032
	Psychosocial fulfillment	35	115, 016
	Educational attainment	7	52, 144
Health-related factors	Good physical health	8	93, 321
	Young age	12	79, 615
Workplace factors	Government/Employer initiatives	12	87, 914
	Job satisfaction Before retirement	4	20, 265
	Job satisfaction in post-retirement work	27	103, 861
Financial factors	Financial necessity	21	95, 479
	Economic context and geographic disparities	2	57, 082
